# Vitamin D Binding Protein and Vitamin D Levels

**DOI:** 10.1155/2014/638263

**Published:** 2014-06-25

**Authors:** Zhongjian Xie, Arthur C. Santora, Sue A. Shapses, Xiangbing Wang

**Affiliations:** ^1^Institute of Metabolism and Endocrinology, The Second Xiangya Hospital, Central South University, Changsha, Hunan 410011, China; ^2^Clinical Research, Diabetes and Endocrinology, Merck Research Laboratories, 126 East Lincoln Avenue, RY34B-148, Rahway, NJ 07065-0900, USA; ^3^Department of Nutritional Science, Rutgers University, New Brunswick, NJ 08901, USA; ^4^Division of Endocrinology, Metabolism and Nutrition, Robert Wood Johnson Medical School, Rutgers University, New Brunswick, NJ 08093, USA

Over the past few decades, there has been a growing interest in understanding the multifunctional characteristics and clinical importance of vitamin D binding protein (DBP). Multiple studies have shed light on DBP, giving rise to hopes of identifying novel mechanisms as well as utilizing DBP as a potential therapeutic agent. The current issue is comprised of 6 manuscripts, two of which are review articles. The areas covered in this special issue mostly highlight the potential role of DBP in several conditions including periodontitis and frailty, implying that measurement of DBP may provide useful information in addition to total 25-hydroxy vitamin D concentration.

In this issue, the review by P. Yousefzadeh et al. titled “*Vitamin D binding protein impact on 25-hydroxyvitamin D levels under different physiologic and pathologic conditions*” calls for the implementation of DBP testing during the interpretation of 25-hydroxyvitamin D [25(OH)D] results under different clinical situations. I. Bhan reviews the recent findings regarding the association between the differences in vitamin D binding protein levels and bone density in his paper titled “*Vitamin D binding protein and bone health.*” The author has compiled studies concerned with DBP, including DBP polymorphisms in relation to bone health and its association with vitamin D bioavailability. These findings may impact not only strategies for designing novel therapeutic agents that influence DBP or its binding, but also our understanding of the mechanism of vitamin D in the context of bone health. Evidence is emerging that the DBP polymorphisms are associated with race and ethnicity, resulting in differences in DBP levels and binding affinity that affect the transport and metabolism of vitamin D and its metabolites.

Using serum samples from various populations with varying DBP levels, J. Freeman et al., in their paper titled “*Influence of vitamin D binding protein on accuracy of 25-hydroxyvitamin D measurement using the ADVIA Centaur vitamin D total assay,*” assessed the agreement between the ADVIA Centaur vitamin D total assay for 25(OH)D testing and the liquid chromatography mass spectrometry (LS-MS/MS) method and concluded that the ADVIA Centaur vitamin D total assay demonstrated good performance compared to the LS-MS/MS method across the normal range of DBP concentrations. This is one example of methods development in the field to ensure accurate and simpler protocols for measuring circulating 25(OH)D in future study.

X. Zhang et al., in their paper titled “*Vitamin D-binding protein levels in plasma and gingival crevicular fluid (GCF) of patients with generalized aggressive periodontitis,*” examined the association of DBP with generalized aggressive periodontitis (GAgP). The authors found that GAgP patients had higher plasma DBP concentrations but lower GCF DBP concentrations than healthy controls, suggesting that decreased GCF-DBP level and increased plasma DBP level are associated with periodontitis. This study adds to the growing evidence of the potential role of DBP in the pathogenesis of periodontitis.

Existing evidence shows an association of high circulating 25(OH)D levels with low TSH levels in younger individuals, but there is insufficient information on whether it is the same in the elderly and middle-aged. Q. Zhang et al., in their paper titled “*Association of high vitamin D status with low circulating thyroid-stimulating hormone independent of thyroid hormone levels in middle-aged and elderly males,*” report that such an association does exist in middle-aged and elderly males, independent of thyroid hormone levels. The authors also demonstrated the link between vitamin D insufficiency and serum thyroid antibody levels.

Y. Wang et al., in their paper entitled “*Vitamin D binding protein affects the correlation of 25(OH)D and frailty in the older men,*” assessed the frailty status in elderly men of Changsha city, China, and concluded that serum DBP levels should be measured when evaluating the 25(OH)D- frailty relationship.

We hope that these articles convey new insights to readers and researchers into the current renewed interest in vitamin D and DBP research.

*Zhongjian Xie* *Zhongjian Xie* *Arthur C. Santora* *Arthur C. Santora* *Sue A. Shapses* *Sue A. Shapses* *Xiangbing Wang* *Xiangbing Wang*

